# Decreasing cerebral oxygen consumption during upright tilt in vasovagal syncope

**DOI:** 10.14814/phy2.13286

**Published:** 2017-05-29

**Authors:** Marvin S. Medow, Mira L. Kothari, Amanda M. Goetz, Mary Breige O'Donnell‐Smith, Courtney Terilli, Julian M. Stewart

**Affiliations:** ^1^ Departments of Pediatrics and Physiology New York Medical College Center for Hypotension Hawthorne New York

**Keywords:** Autonomic nervous system, near‐infrared spectroscopy, orthostatic intolerance, transcranial doppler ultrasound

## Abstract

We measured changes in transcranial Doppler ultrasound (TCD) and near infrared spectroscopy (NIRS) during 70° upright tilt in patients with recurrent vasovagal syncope (VVS,* N* = 20), postural tachycardia syndrome (POTS,* N* = 20), and healthy controls (*N* = 12) aged 15–27 years old. VVS was included if they fainted during testing within 5–15 min of upright tilt. We combined TCD and NIRS to obtain estimates of percent change in the cerebral metabolic rate of oxygen consumption (CMRO_2_), cerebral blood flow velocity (CBFv), and oxygen extraction fraction (OEF). Over the course of 10 min of upright tilt, CBFv decreased from a baseline of 70 ± 5 to 63 ± 5 cm/sec in controls and 74 ± 3 to 64 ± 3 cm/sec in POTS while decreasing from 74 ± 4 to 44 ± 3 cm/sec in VVS. CMRO_2_ was unchanged in POTS and controls during tilt while OEF increased by 19 ± 3% and 15 ± 3%, respectively. CMRO_2_ decreased by 31 ± 3% in VVS during tilt while OEF only increased by 7 ± 3%. Oxyhemoglobin decreased by 1.1 ± 1.3 *μ*mol/kg brain tissue in controls, by 1.1 ± 1.3 *μ*mol/kg in POTS, and 11.1 ± 1.3 *μ*mol/kg in VVS. CBFv and CMRO_2_ fell steadily in VVS during upright tilt. The deficit in CMRO_2_ in VVS results from inadequate OEF in the face of greatly reduced CBF.

## Introduction

Syncope (fainting) is defined by a rapid onset of transient loss of consciousness and postural tone due to global cerebral hypoperfusion with spontaneous recovery (Folino et al. 2007). Systemic hypotension is present as the precipitant cause. VVS causes loss of postural tone, which promotes physical injury and rarely accidental death. The most common form of syncope in the young is simple postural faint, denoted “postural vasovagal syncope” (VVS) (Gowers 1907), and is a common form of orthostatic intolerance (OI). Prodromal (presyncopal) symptoms often include lightheadedness, cognitive deficits, visual loss headache, sweating, tremulousness, hyperventilation, and a sensation of heat presaging imminent syncope (Low et al. 1995a) which relate to deficient cerebral perfusion, sympathoadrenal activation, and perisyncopal vasodilation. Indeed, an abnormal orthostatic decrease in cerebral blood flow preceding overt syncope has been well documented (van Lieshout et al. 2003; Hermosillo et al. 2006). The diagnosis of VVS depends strongly on medical history including the characteristic prodrome, appearance during the faint and postdrome, and also by ruling out cardiac causes of syncope (Sheldon et al. 2015). Simple faint is most often episodic and associated with long periods of “wellness” (Stewart 2009).

In contrast, postural tachycardia syndrome (POTS) identifies with day‐to‐day chronic orthostatic intolerance (Schondorf and Low 1993; Low et al. 1995b). POTS is defined in adults by OI symptoms – lightheadedness, cognitive deficits, headache, sweating, tremulousness, but absent visual loss (blackout) or the presyncopal sensation of heat due to vasodilation – along with an excessive increase in heart rate exceeding 30 beats/min within 10 min of orthostasis in the absence of postural hypotension (Schondorf and Low 1993). BP may even increase (Grubb and Kosinski 1997). Larger HR orthostatic increments (~40 bpm) in association with chronic OI symptoms in the absence of postural hypotension are needed to make the diagnosis of POTS in the young (Stewart et al. 2006; Singer et al. 2012). Most often, mean cerebral blood flow when upright decreases gradually similar to healthy volunteers (Zhang et al. 1985). Excessively reduced cerebral blood flow has been observed in some POTS patients during orthostatic challenge, but it is an inconstant finding (Del Pozzi et al. 2014). When orthostatic stress is gradual during incremental upright tilt, cerebral blood flow decreases similarly in POTS and control (Stewart et al. 2012). For this reason POTS is unassociated with a sudden loss of consciousness except in a minority of POTS patients (~15–30%) who also have episodes of VVS (Sheldon et al. 2015).

The human brain is one of the most metabolically active organs in the body and consumes approximately 3.5 mL of O_2_ per 100 g of brain tissue per minute varying with specific brain location and tissue type (Lassen 1959). Severe global cerebral ischemia decreases the cerebral metabolic rate of oxygen consumption (CMRO_2_) and aerobic metabolism causing loss of cerebral function and loss of consciousness (Tsivgoulis and Alexandrov 2008).

We hypothesized that reduced CMRO_2_ during orthostasis would occur in VVS because it is identified with global cerebral hypoperfusion and would not occur in POTS because cerebral blood flow is preserved.

## Materials and Methods

To address this hypothesis we combined near‐infrared spectroscopy (NIRS) with transcranial Doppler ultrasound (TCD) in VVS patients, POTS patients, and healthy volunteer control subjects.

### Subjects

Twenty patients with a history of recurrent VVS aged 21.2 ± 1.2 years (13 females), with 3 or more episodes within the past 12 months, 20 POTS patients aged 22.5 ± 2.0 years (16 females), and 12 healthy volunteers aged 22.0 ± 1.1 years (8 females) ranging in age from 15 to 27 years old were recruited. There were no group differences in age range, weight, or body mass index. No subjects were taking neurally active or vasoactive drugs. Exclusionary criteria for all subjects included any infectious or systemic disease, including diseases of the central nervous system, any other well‐characterized autonomic disease, any endocrine, respiratory, metabolic, or cardiovascular diseases, competitive athletic training, recent long‐term bed rest, the use of nicotine or any other chronic medication; excluding oral contraceptives.

### Healthy volunteer control subjects

Healthy volunteer control subjects were recruited from adolescents and young adults recruited as control subjects for unrelated NIH studies. Control subjects with a history of syncope or orthostatic intolerance were specifically excluded. Volunteers were retained if they did not faint during the tilt test.

### Vasovagal syncope

Patients with Vasovagal syncope (VVS) were referred to our center for investigation after experiencing at least three episodes of fainting within the last 12 months. The diagnosis of VVS was primarily based on the clinical history. Key diagnostic features included predisposing situations, prodromal symptoms, physical signs, and postdrome recovery and symptoms (Sheldon et al. 2015). In all patients, past fainting had been induced by prolonged standing and in three patients it was also triggered by noxious stimuli. Prodromal features included pallor, lightheadedness, nausea with abdominal discomfort, diaphoresis usually followed by a feeling of warmth, visual scotomata, or frank loss of vision progressing toward loss of consciousness. By design VVS patients were recruited if they had recurrent syncope and had postural vasovagal syncope in the laboratory in which symptoms and signs similar to their real‐world syncope were reproduced without pharmacological potentiation. Unconsciousness lasted less than 30 sec in all VVS patients once placed supine and most patients felt profoundly fatigued afterward. Prior medication for syncope, if any, was stopped for at least 2 weeks prior to participation in this study. Although VVS patients had recurrent episodes of syncope they lacked chronic day‐to‐day OI symptoms.

### Postural tachycardia syndrome

Potential POTS patients for the study were selected based on referral to our clinic for chronic orthostatic intolerance lasting longer than 6 months; diagnosis was confirmed in our laboratory by means of a screening 70° upright tilt test (HUT). The criteria used for POTS in patients over 19 years old were a heart rate (HR) increase exceeding 30 bpm or absolute HR exceeding 120 bpm within 10 min of tilt to 70° associated with OI symptoms in the absence of hypotension (Freeman et al. 2011). The criteria used for POTS in adolescents <19 years old were an increase in HR exceeding 40 bpm or absolute HR exceeding 120 bpm during a 10 min tilt to 70^o^ in the absence of hypotension (Singer et al. 2012). POTS enrollees were either therapeutically naïve or were weaned off medication except for oral contraceptives 2 weeks before studies. POTS subjects experiencing syncope during upright tilt were excluded from participation. No patients were perimenstrual at the time of study.

During an upright tilt test of VVS, if reflex tachycardia were excessive, erroneous diagnoses of POTS are made because POTS is chronic orthostatic intolerance, while episodic VVS is not. While a diagnosis of syncope by laboratory testing requires a finding of hypotension, this specifically excludes a diagnosis of POTS. Excessive tachycardia preceding hypotension and bradycardia is often part of the vasovagal response, and can be observed during tilt table testing of patients diagnosed with VVS, who are free of chronic day‐to‐day symptoms of OI, but do have postural hypotension and therefore do not have POTS. We have recently shown that HR increases alone do not confer a diagnosis of POTS (Pediatrics Reference), thus our cohort of POTS and VVS patients each represent a unique population. JULIAN, one of the major criticisms was due to confusion based on the increased HR after HUT in VVS causing the reviewer to say “they have POTS”. Perhaps you can add the PEDIATRICS paper as a new citation to resolve this confusion.

The study was approved by the Committee for the Protection of Human Subjects (Institutional Review Board) of New York Medical College conforming to The Declaration of Helsinki. All subjects 18 years and older signed informed consent prior to participation and those <18 years old gave assent and their legal guardians signed informed consent forms.

### Instrumentation

#### Cardiopulmonary monitoring

Participants arrived for testing at 9:30 am after a 2 h fast. Participants were instructed about the experiment and were instrumented. Instrumentation included a beat‐to‐beat arterial blood pressure finger plethysmograph (Finometer, FMS, Amsterdam, The Netherlands) calibrated against oscillometric BP. Respiratory plethysmography (Respitrace 200, NIMS Inc., North Bay Village Florida) was used to measure changes in respiration. A combined capnograph and integrated pulse oximeter (Smith Medical PM, Waukesha, WI) was used to measure end‐tidal carbon dioxide (ETCO_2_) and arterial oxygen saturation. HR was measured from the beat‐to‐beat cardiac electrical intervals on an electrocardiogram. We used the ModelFlow pulse contour algorithm of the Finometer calibrated against an Innocor inert gas rebreathing cardiac output at baseline (Innovision, Denmark) to obtain relative values for cardiac output (CO) and total peripheral resistance (TPR) calculated as MAP/CO.

### Transcranial Doppler Ultrasound and near‐infrared spectroscopy

A 2 MHz transcranial Doppler (TCD) (Multigon, Yonkers, NY) insonated the left middle cerebral artery (MCA) with the signal optimized for depth and signal strength. TCD was used to measure changes in cerebral blood flow velocity (CBFv). Detection of middle cerebral artery blood flow was adjusted to measure the maximum value of CBFv ensuring that the area of pulse wave interrogation was centered in the artery. All measurements were monitored continuously to assure that there were no sudden positional changes during the performance of these studies.

A continuous‐wave spatially resolved near‐infrared spectrometer (NIRS) (Oxymon MKII, Artinis) was used to monitor changes in oxygenated hemoglobin (HbO_2_), deoxyhemoglobin (Hb), and Total hemoglobin (THb) over the volume of cerebral tissue insonated by TCD throughout the protocol (Rasmussen et al. 2007). Subjects were instrumented with an emitter and a detector pair of NIRS probes on the forehead over the right frontal cortex to monitor absorption of light across the cerebral frontal area that is primarily perfused by the MCA (Ross et al. 2014). Infrared signals produced by the cutaneous bed were resolved through spatial resolution insuring that the infrared signals from the brain were the only signals analyzed. NIRS was sampled at 50 Hz. Using an integrated digital to analog converter, the sampled NIRS signal was reconstructed as an analog signal and then resampled. The modified Beer‐Lambert law was used to calculate micromolar changes in tissue HbO_2_ and Hb across time using optical densities from near‐infrared light at 780 and 850 nm. Depending upon the subject characteristics different differential path‐length factors were used for each individual participant (Essenpreis et al. 1993). The volume of cerebral tissue illuminated may differ between the participants; however, within each participant the volume of cerebral tissue was assumed constant for the duration of the experiment. In order to index changes in blood volume within the illuminated brain volume, changes in THb were obtained by adding the changes in HbO_2_ to the changes in Hb. The averages of Hb and HbO_2_ during quiet rest were used to define baseline values.

Because these are noninvasive measurements, we made the assumption that percent changes in CBFv estimated percent changes in cerebral blood flow in the conduit vessels feeding the frontal cortical tissue that was illuminated by the NIRS. fMRI data suggest that MCA diameter is constant in response orthostatic stress (Serrador et al. 2000). Most important for this investigation, literature also indicates that percent change in CBFv in the MCA can be used to accurately estimate percent change in cerebral blood flow in the MCA (Ainslie and Hoiland 2014). Accordingly, CBFv changes are depicted graphically as percent change and normalized to baseline values obtained for each participant.

All analog signals were digitized at 200 Hz with custom signal‐processing software and were analyzed offline by the same trained researcher after completion of studies in all subjects.

### Protocol

After instrumentation, subjects remained supine for 30 min to acclimate to their surroundings and the equipment. Following the 30 min acclimation period, a 10 min baseline period was recorded.

After supine measurements were complete, POTS and control patients were tilted to 70° for 10 min; VVS patients may have been tilted slightly longer if necessary. For purposes of comparison we retained VVS patients who had tilt‐induced VVS between 5 and 15 min after tilt up. An electrically driven tilt table (Cardiosystems 600, Dallas, Texas) with a footboard was used. Changes in cerebral oxyhemoglobin (HbO_2_) and deoxyhemoglobin (Hb) concentrations were measured by NIRS while CBFv of the MCA was measured by TCD. A priori stopping criteria for fainting was defined by a rapid decrease in systolic blood pressure (SBP) to <60 mmHg or a decrease in SBP to <80 mmHg associated with symptoms of impending loss of consciousness, severe lightheadedness, heat, nausea, or diaphoresis.

### Timed data collection

Orthostasis immediately causes rapid translocation of an estimated 500 mL of central blood volume caudally due to gravity. There is a transient loss of mechanical (pressure–volume) equilibrium caused by a brief lag (~10 sec) in the onset of neurovascular compensation; a transient decrease in BP and reflex increase in HR occurs. Recovery of BP and mechanical equilibrium occurs within a minute, and usually in <30 sec. Such transient hypotension is denoted “initial orthostatic hypotension” (IOH), a frequent but brief response to orthostatic stress (Wieling et al. 2007). Afterward BP stabilizes at systolic and diastolic BP typically slightly elevated from baseline. BP usually remains stable while HR progressively increases in POTS and also in healthy volunteers typically reaching an asymptote between 5 and 10 min. By definition, POTS patients, but not control subjects, have excessive tachycardia and OI symptoms as defined above. In contrast, VVS patients, as described by Julu et al. (2003) also have BP stability which is often followed by a prolonged intermediate phase during which BP gradually decreases while HR increases. Thereafter, both HR and BP fall precipitously with the onset of fainting (Stewart 2013). For purposes of inter‐Group comparison we collected time averaged data at supine baseline, 1 min after tilt (once mechanical equilibrium is achieved), 5 min after tilt, at 10 min after tilt, and at the end of tilt which coincided with the onset of faint in VVS patients. If fainting occurred in <10 min we used the data at the initiation of faint in place of 10 min data.

### Data analysis

Mean arterial Pressure (MAP) was calculated from systolic (SBP) and diastolic BP (DBP) as: MAP = (1/3)·SBP + (2/3)·DBP. Mean CBFv was calculated from systolic CBFv and diastolic CBFv as: mean CBFv = (1/3)·systolic CBFv + (2/3)·diastolic CBFv. MAP and mean CBFv were verified by automated multibeat time averaging.

NIRS generates relative changes in concentrations of HbO_2_ and Hb (designated ΔHb and ΔHbO_2_ that when summed yields ΔTHb). As shown by Mayhew, Jones, Zheng, and coworkers, capillary and tissue oxygen profiles rapidly reach a steady state dependent on capillary transit time (~0.2 sec) and parenchymal oxygen diffusion (Zheng et al. 2002, 2005). CBFv, ΔHb, ΔHbO_2_, arterial, and venous hemoglobin saturations change relatively slowly compared with tissue oxygen profiles permitting the use of steady‐state kinetics to estimate time varying CMRO_2_ such that (Ogawa et al. 1993; Jones et al. 2001; Mayhew et al. 2001; Boas et al. 2003)

(1) $$\begin{array}{cc} & \left(\frac{\Delta {\text{CMRO}}_{2}+{\text{CMRO}}_{2}}{{\text{CMRO}}_{2}}\right)\\ & =\left(\frac{\Delta {\text{Hb}}_{v}+{\text{Hb}}_{v}}{{\text{Hb}}_{v}}\right)⨂{\left(\frac{\Delta {\text{THb}}_{v}+{\text{THb}}_{v}}{{\text{THb}}_{v}}\right)}^{-1}\left(\frac{\Delta \text{CBF + CBF}}{\text{CBF}}\right) \end{array}$$

where the subscript *v* denotes the venous compartment. This approach was recast and validated in humans by Boas et al. (2003) as:

(2) $$\left(1+\frac{\Delta {\text{CMRO}}_{2}}{\;{\text{CMRO}}_{2}}\right)=\frac{\left(1+\frac{\Delta \text{Hb}}{\text{Hb}}\right)⨂\left(1+\frac{\Delta \text{CBF}}{\text{CBF}}\right)}{\left(1+\frac{\Delta \text{THb}}{\text{THb}}\right)}$$

where ΔHb, ΔHbO_2_, ΔTHb now refer to changing hemoglobin per unit mass of cerebral cortex. Over an infinitesimal time interval Δ*t*, ΔCMRO_2_ << CMRO_2,_ ΔHb<< Hb, ΔTHb<<THb, and all changes approach infinitesimals. Using a Taylor series and discarding second‐order terms (Apostol 1991)

(3) $$\frac{1}{\left(1+\frac{\Delta \text{THb}}{\text{THb}}\right)}\approx \left(1-\frac{\Delta \text{THb}}{\text{THb}}\right)$$

and therefore

(4) $$\begin{array}{cc} & \left(1+\frac{d{\text{CMRO}}_{2}}{{\text{CMRO}}_{2}}\right)\\ & \approx \left[\left(1+\frac{d\text{Hb}}{\text{Hb}}\right)\left(1+\frac{d\text{CBF}}{\text{CBF}}\right)\left(1-\frac{d\text{THb}}{\text{THb}}\right)\right] \end{array}$$

where ‘*d*’, indicating an infinitesimal, has replaced ‘Δ’.

Assuming cross terms (i.e., $\frac{dx}{x}\cdot \frac{dy}{y}$) are vanishingly small then

(5) $$\left(1+\frac{dCMRO_{2}}{CMRO_{2}}\right)\approx 1+\frac{dHb}{Hb}+\frac{dCBF}{CBF}-\frac{dTHb}{THb}$$

where all terms are time dependent. Subtracting 1 from both sides and introducing the time infinitesimal *dt*, we get

(6) $$\left(\frac{1}{{\text{CMRO}}_{2}}\frac{d{\text{CMRO}}_{2}}{dt}\right)\approx \frac{1}{\text{Hb}}\frac{d\text{Hb}}{dt}+\frac{1}{\text{CBF}}\frac{d\text{CBF}}{dt}-\frac{1}{\text{THb}}\frac{d\text{THb}}{dt}$$

Or

(7) $$\left(\frac{d\ln\left({\text{CMRO}}_{2}\right)}{dt}\right)\approx \frac{d\ln\left(\text{Hb}\right)}{dt}+\frac{d\ln\left(\text{CBF}\right)}{dt}-\frac{d\ln\left(\text{THb}\right)}{dt}$$

And integrating

(8) $$\ln\left(\frac{{\text{CMRO}}_{2}\left(t\right)}{{\text{CMRO}}_{2}\left(0\right)}\right)\approx \ln\left(\frac{\text{Hb}\left(t\right)}{\text{Hb}\left(0\right)}\right)+\ln\left(\frac{\text{CBF}\left(t\right)}{\text{CBF}\left(0\right)}\right)-\ln\left(\frac{\text{THb}\left(t\right)}{\text{THb}\left(0\right)}\right)$$

where ln denotes the natural logarithm.

The fractional change in CMRO_2_ was obtained by taking the exponential of the right hand side of eq. (9) **.** We used data from the literature to estimate Hb(0) = 35 *μ*mol/L per kg‐tissue and THb(0) = 100 *μ*mol/L per kg‐tissue over the cerebral tissue as per Boas et al. (2003). The Oxygen Extraction Fraction (OEF) = CMRO2/(CBF) can be calculated from

(9) $$\left(\frac{\text{OEF}\left(t\right)}{\text{OEF}\left(0\right)}\right)=\left(\frac{{\text{CMRO}}_{2}\left(t\right)}{{\text{CMRO}}_{2}\left(0\right)}\right)÷\left(\frac{CBF\left(t\right)}{\text{CBF}\left(0\right)}\right)$$

(Uludag et al. 2004)

Resting values of OEF are typically ~0.4 (Raichle et al. 2001).

### Statistics

Because there were no significant differences in results between the sexes, both male and female data were combined for group analysis. During baseline and upright tilt the measurements of arterial pressures, HR, CO, TPR, ETCO_2_, CBFv via TCD, changes in Hb, HbO_2_, and THb via NIRS, and estimated fractional changes in CMRO_2_ and in OEF were measured or calculated. For CMRO_2_, we measured the change in light reflected back following illumination from a constant source that informs on the relative proportion of oxygenated/deoxygenated hemoglobin. Thus, the relative proportion of reflected light (i.e., the percent change) is the unit of analysis. During tilt, we represented CBFv, MAP, CMRO_2_, and OEF as percent changes from baseline.

We used a repeated‐measures ANOVA with two factors: study group (at two levels) and period (at four or five levels, depending on the outcome). We were interested in a GROUP x PERIOD interaction, in which a significant interaction would indicate that the study groups differ at one or more of the periods. In the case where the assumption of sphericity is violated, a Greenhouse‐Geisser correction to the degrees of freedom of the *F*‐test was used.

We compared healthy volunteer control subjects, with VVS patients and POTS patients at baseline, 1 min post tilt, 5 min post tilt, and 10 min post tilt by two‐way ANOVA with time from tilt as a repeated measure looking for Group x Time differences. Significance was set a priori and differences were considered significant when a *P *<* *0.05 was achieved. All values are reported as means ± SEM. Results were calculated using SPSS 16 (SPSS Inc., Chicago IL).

## Results

By design all the VVS patients developed a vasovagal faint while upright, with hypotension and bradycardia at the onset of fainting. None of the POTS or control subjects had hypotension or syncope (Freeman et al. 2011).

### Baseline supine results (Table 1)

**Table 1:** Baseline and upright tilt characteristics

	Baseline	1 min	5 min	10 min	Faint
Systolic BP (mmHg)					
Control	116 ± 4	122 ± 4	119 ± 3	121 ± 4	119 ± 3
VVS^a^	115 ± 2	118 ± 4	120 ± 2	109 ± 2	63 ± 6
POTS	118 ± 3	124 ± 3	126 ± 3	124 ± 2	125 ± 2
Diastolic BP (mmHg)					
Control	56 ± 2	63 ± 3	66 ± 3	64 ± 2	63 ± 2
VVS^a^^,^ b	57 ± 2	67 ± 4	64 ± 3	54 ± 2	33 ± 3
POTS^a^	61 ± 2	68 ± 2	71 ± 2	70 ± 2	71 ± 2
Mean BP (mmHg)					
Control	76 ± 3	83 ± 3	84 ± 2	83 ± 2	84 ± 2
VVS^a^^,^ b	76 ± 2	84 ± 3	83 ± 2	73 ± 2	43 ± 2
POTS	80 ± 2	87 ± 2	89 ± 2	88 ± 2	89 ± 4
HR (bpm)					
Control	66 ± 3	79 ± 5	82 ± 5	81 ± 5	83 ± 5
VVS^a^^,^ b	67 ± 2	96 ± 4	103 ± 4	108 ± 5	69 ± 3
POTS^a^	73 ± 3	118 ± 5	122 ± 5	123 ± 5	125 ± 5
ETCO_2_ (Torr)					
Control	40 ± 1	38 ± 1	38 ± 1	38 ± 1	38 ± 1
VVS^a^	43 ± 1	39 ± 1	37 ± 2	34 ± 2	31 ± 1
POTS	43 ± 1	37 ± 1	36 ± 1	35 ± 1	35 ± 1
Cardiac output (L/min)					
Control	5.6 ± .0.4	5.2 ± .0.4	5.3 ± 0.4	5.3 ± 0.4	5.3 ± 0.4
VVS	5.0 ± .0.4	4.7 ± .0.4	4.7 ± 0.3	4.4 ± 0.4	2.8 ± 0.5
POTS	5.1 ± .0.4	4.6 ± .0.4	4.7 ± 0.3	4.6 ± 0.3	4.6 ± 0.3
Total peripheral resistance (mmHg/L/min)					
Control	13 ± 2	17 ± 1	17 ± 1	16 ± 1	17 ± 1
VVS^a^^,^ b	14 ± 1	20 ± 1	17 ± 1	14 ± 2	13 ± 2
POTS^a^	17 ± 1	20 ± 1	20 ± 1	20 ± 1	21 ± 1
CBFv (cm/sec)					
Control	70 ± 5	66 ± 6	64 ± 5	63 ± 5	63 ± 5
VVS^a^^,^ b	74 ± 4	65 ± 3	57 ± 3	52 ± 3	44 ± 3
POTS	74 ± 3	65 ± 4	64 ± 3	65 ± 3	64 ± 3
ΔHbO_2_ (*μ*mol kg/tissue)					
Control	0.0 ± 0.0	−1.9 ± 0.9	−1.1 ± 1.2	−0.7 ± 1.3	−.1 ± 1.3
VVS^a^	0.0 ± 0.0	−5.6 ± 0.9	−6.5 ± 1.3	−7.5 ± 1.2	−11.1 ± 1.3
POTS^a^	0.0 ± 0.0	−3.2 ± 1.2	−3.1 ± 1.3	−2.8 ± 1.3	−2.7 ± 1.3
ΔHb (*μ*mol kg/tissue)					
Control	0.0 ± 0.0	1.7 ± .0.7	2.3 ± 0.5	2.2 ± 0.5	2.4 ± 0.5
VVS^a^^,^ b	0 ± 0	−.1 ± 0.5	−0.2 ± 0.7	−0.2 ± 0.8	0.8 ± 1.3
POTS	0 ± 0	1.7 ± 0.6	2.1 ± 0.6	2.3 ± 0.6	2.4 ± 0.6
ΔTHb (*μ*mol kg/tissue)					
Control	0.0 ± 0.0	−0.2 ± 0.7	1.2 ± 0.8	1.5 ± 0.9	2.27 ± 0.8
VVS^a^^,^ b	0.0 ± 0.0	−5.7 ± 1.4	−6.7 ± 1.8	−7.6 ± 1.9	−10.1 ± 2.4
POTS	0.0 ± 0.0	−1.5 ± 1.7	−1.0 ± 1.8	−0.5 ± 1.8	−0.3 ± 1.7

CBFv, cerebral blood flow velocity; VVS, vasovagal syncope; POTS, postural tachycardia syndrome.

a Significantly different from control.

b Significantly different from POTS.

Systolic, diastolic, and mean arterial pressures were not different at baseline while supine. At baseline, HR was higher in POTS compared to control (*P* < 0.05), but the same as VVS. Neither CBFv nor CO differed by group. ETCO_2_ was decreased for both VVS (*P* < 0.005) and for POTS (*P* < 0.025) compared to control, but did not differ from one another.

### Upright measurements

**Figure 1:**
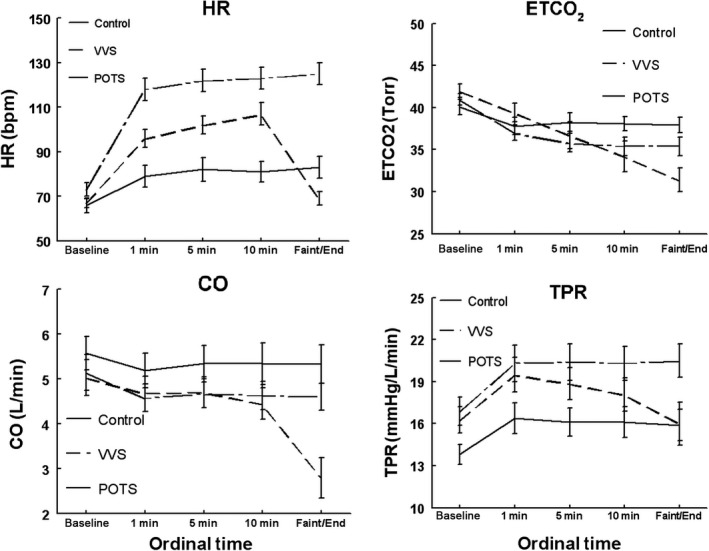
Heart rate (HR), end tidal carbon dioxide (ETCO_2_), cardiac output (CO) and total peripheral resistance (TPR) during upright tilt for control subjects (solid line), vasovagal syncope patients (VVS, dashed line) and postural tachycardia syndrome patients (POTS, dash and dot line). HR is most increased above control in POTS and to a lesser degree in VVS. TPR is increased in POTS compared to control, and falls throughout tilt in VVS.

**Figure 2:**
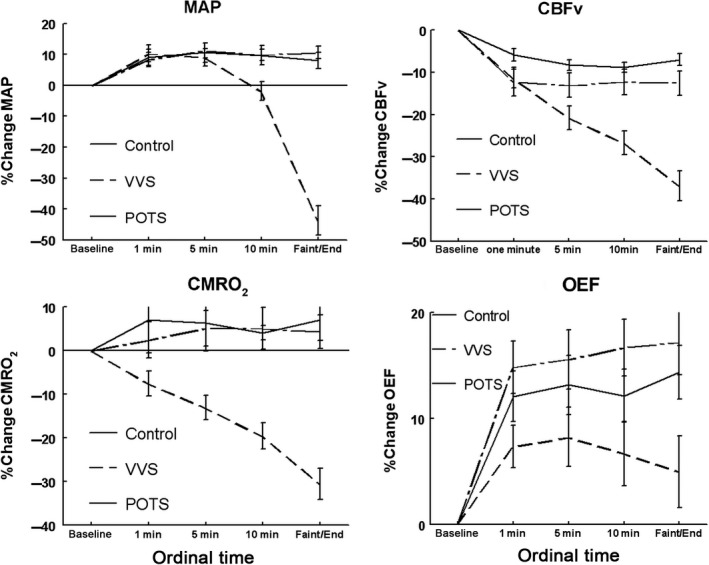
Percent change mean in arterial pressure (MAP), in cerebral blood flow velocity (CBFv), in cerebral metabolic rate of oxygen consumption (CMRO_2_) and in oxygen extraction fraction (OEF) during upright tilt for control subjects (solid line), vasovagal syncope patients (VVS, dashed line) and postural tachycardia syndrome patients (POTS, dash and dot line). MAP declines in VVS but is stable in control and POTS. CBFv and CMRO2 decline monotonically and in parallel throughout tilt in VVS. OEF increases by approximately half as much as control in VVS. CBFv decreases equally in control and POTS, while CMRO_2_ is well maintained.

These are shown in the Table 1, and are depicted in Figures 1 and 2.

### HR, ETCO2, CO, and TPR during upright tilt (Fig. 1)

HR was significantly increased above control in POTS (*P* < 0.001) and in VVS (*P* < 0.01). These differences persisted even if the final data points (denoted faint/end) were removed. Averaged HR in VVS was significantly smaller than the averaged HR in POTS (*P* < 0.01).

ETCO_2_ decreased in all groups during tilt but was significantly decreased for VVS (*P* < 0.05) compared to control.

CO was not different for VVS except for the actual faint. There was a tendency for a group effect for both VVS and POTS compared to control which did not reach significance (*P* = 0.16).

TPR increased initially during tilt for all groups, but was thereafter progressively reduced only for VVS (Group•Time *P* < 0.001). TPR was elevated for POTS compared to control and maintained a significant group difference from control throughout tilt (*P* < 0.001).

### % Change in MAP, CBFv, CMRO_2_, and OEF during upright tilt (Fig. 2)

MAP initially increased upon tilt in all groups and remained, on average, unchanged in POTS and control for the remainder of upright tilt. MAP fell throughout tilt in VVS (*P* < 0.001).

CBFv decreased from baseline for all groups (*P*_time_ < 0.01). The time course was not different for control and POTS and tended to asymptote to a 10% reduction. In contrast, CBFv was progressively reduced throughout tilt for VVS (*P* < 0.001).

CMRO_2_ was not different from baseline for control and POTS. CMRO_2_ progressively decreased in VVS throughout tilt (*P* < 0.001).

OEF increased from baseline in all subjects and was not different in POTS compared to control subjects. OEF was significantly reduced in VVS compared to control and to POTS (both *P* < 0.01) indicating reduced oxygen extraction in VVS in conjunction with decreased CBFv. Reduced CBFv is not compensated by a reciprocal increase in OEF and thus CMRO_2_ falls.

## Discussion

Our new findings are that CBFv and cerebral metabolic rate of oxygen consumption (CMRO_2_) fall steadily in VVS during upright tilt. The deficit in CMRO_2_ results from reduced oxygen extraction fraction (OEF) and reduced CBF. OEF does not increase commensurate with the reduction in CBFv in VVS. CBFv, CMRO_2,_ and OEF in POTS do not differ from control. OEF increases sufficiently in POTS and control subjects to compensate for the decrease in CBFv.

Orthostatic intolerance includes symptoms imputed to diminished central nervous system function such as impaired memory and thought, central fatigue, lightheadedness, and headache. These symptoms are common to both VVS and POTS. In VVS lightheadedness and confusion give way to loss of consciousness as hypotension‐bradycardia supervene. This does not occur in POTS because hypotension is absent.

Based on our data the progression of CNS impairment in VVS is easily understood: there is a marked and early loss of cerebral blood flow and early reduction in CMRO_2_ due to reduced compensatory oxygen extraction.

CMRO_2_ is lower in VVS than in control early during orthostatic stress. One explanation for decreased OEF is that tissue oxygen is reduced to low values in VVS during early orthostatic stress. This would maximize the vascular to parenchyma oxygen gradient permitting the maximum rate of oxygen consumption under such conditions.

Alternatively, an abnormal reduction in neural function would reduce aerobic metabolism in VVS patients during orthostatic stress limiting vascular to parenchyma oxygen extraction and OEF.

CMRO_2_, CBFv, and OEF are similar in POTS and control subjects. This despite ongoing orthostatic cognitive impairment in POTS (Ocon 2013; Ross et al. 2014), sometimes referred to as ‘brain fog’ (Ross et al. 2013). In past work, we used a working memory task in younger POTS patients to demonstrate this progressive cognitive impairment during step‐wise incremental orthostatic stress (Ocon et al. 2012). Cognitive impairment in POTS was not associated with excessively decreased CBFv compared to control subjects but rather with defective neurovascular coupling (NVC) (Stewart et al. 2012) such that neuronal task related increases in cerebral blood flow (functional hyperemia) was absent in POTS patients (Stewart et al. 2012). NVC is the relationship between neural activity and functional hyperemia and involves interactions among components of the neurovascular unit. Further study attributed loss of NVC in POTS to oscillatory cerebral blood flow rather than a decrease in mean CBF (Stewart et al. 2015, 2016). Disruption of NVC has been shown in diabetes, depression, hypertension, stroke, and Alzheimer's disease. However, the relationship between NVC and cerebral oxygen consumption has yet to be determined (Stewart et al. 2012).

A substantive decrease in CBF occurs in postural vasovagal syncope, impairs cerebral oxygenation, and causes prodromal lightheadedness and cognitive loss (brain fog) ultimately producing loss of consciousness, perhaps the acme of “brain fog”.

### Limitations

POTS patients contained a larger percent of females than VVS or control groups. There were no qualitative differences in results based on gender and male and female subjects were pooled.

This study is limited by the use of TCD and NIRS which are indirect methods for the estimation of changes in CBF, and they have low regional specificity. However, postural VVS is a dynamic upright time‐dependent process and TCD + NIRS have superior time resolution compared with, say fMRI or PET scans, and can be easily applied during upright tilt. Use of more sophisticated imaging tools is precluded because of the experimental arrangement, changing position, and constant variations of cerebral perfusion and oxygenation.

Most studies utilizing TCD to measure CBFv within cerebral conduit vessels assume that the MCA is approximately uniform and cylindrical. In similar experiments this assumption has served well as a useful approximation (Aaslid et al. 2003). Results depend critically on how well Doppler estimates of CBFv reflect changes in cerebral blood flow during orthostatic stress. This, in turn, depends on whether the MCA diameter remains unchanged during orthostasis. fMRI data suggest that MCA diameter is unchanged with orthostatic stress due to LBNP (Serrador et al. 2000). Recent literature also indicates that percent change in CBFv in the MCA can be used to estimate percent change in CBF during orthostatic challenge with a 2% bias overall (Lewis et al. 2015). As changes in CBFv (ΔCBFv) are indexed to CBFv during calculations of ΔCMRO_2_/CMRO_2_ our assumptions should be valid. We also assumed that changes in TCD CBFv correspond primarily to changes in the flow velocity of the middle cerebral artery that also supplies blood to the area of NIRS illumination (Rasmussen et al. 2007). Finally, we assumed that time‐dependent changes in CMRO_2_, CBFv, ΔHb, and ΔHbO2 are slow with respect to equilibration between vascular and parenchymal oxygenation. Under the conditions of this study these assumptions seem reasonable (Stewart et al. 2006, 2012).

The ModelFlow method for computing cardiac output has shortcomings. While there are no studies specifically comparing ModelFlow cardiac output calculations to, say, thermodilution catheter computations in patients with presyncope or syncope, there are studies in which comparisons were made in intensive care patients showing acceptable errors without consistent bias (Schloglhofer et al. 2014).

## Conflict of Interest

None declared.
